# Aminothiol supported dialdehyde cellulose for efficient and selective removal of Hg(II) from aquatic solutions

**DOI:** 10.1038/s41598-023-46082-3

**Published:** 2023-11-09

**Authors:** Aya G. Mostafa, Eslam A. Gaith, Magda A. Akl

**Affiliations:** Department of Chemistry, Faculty of Science, Mansoura University, Mansoura, 35516 Egypt

**Keywords:** Analytical chemistry, Environmental chemistry, Materials chemistry, Organic chemistry, Polymer chemistry

## Abstract

The increasingly serious problem of mercury pollution has caused wide concern, and exploring adsorbent materials with high adsorption capacity is a simple and effective approach to address this concern. In the recent study, dialdehyde cellulose (DAC), cyanoacetohydrazide (CAH), and carbon disulfide (CS_2_) are used as raw materials for the (DAC@CAH@SK_2_) preparation material through the three-steps method. By utilizing the following characterization techniques; thermogravimetric analysis (TGA), N_2_ adsorption–desorption isotherm (BET), elemental analysis, scanning electron microscopy (SEM), Fourier transform infrared spectroscopy (FTIR), and X-ray diffraction (XRD), ^1^HNMR and Energy Dispersive X-ray Spectroscopy (EDS) of DAC@CAH@SK_2_ composite. The point of zero charge (pH_PZC_) for the prepared DAC@CAH@SK_2_ also was examined. From the batch experiments, the optimum conditions were found to be pH (5–8), an Hg^2+^ concentration of 150 mg/L, a DAC@CAH@SK_2_ dose of 0.01 g, and a contact time of 180 min with a maximum adsorption quantity of 139.6 mg/g. The process of Hg^2+^ adsorption on the DAC@CAH@SK_2_ material was spontaneous exothermic, monolayer chemisorption, and well-fitted to Langmuir and pseudo-2nd-order models. The DAC@CAH@SK_2_ selectivity towards the Hg^2+^ was examined by investigating the interfering metal ions effect. The DAC@CAH@SK_2_ was successfully applied for the Hg^2+^ removal from synthetic effluents and real wastewater samples with a recovery % exceeding 95%. The prepared DAC@CAH@SK_2_ was regenerated using a mixture of EDTA and thiourea. Also, FT-IR analysis indicates that the synergistic complexation of N and S atoms on DAC@CAH@SK_2_ with Hg(II) is an essential factor leading to the high adsorption capacity.

## Introduction

The rapid growth of industry, population, and urbanization causes significant water contamination^1,2^. Mercury(II) is considered an extremely toxic contaminant that can easily accumulate in the human body^3^. It poses a dangerous threat to the environment and living organisms' health as it causes such serious illnesses as paralysis, birth defects, mental disorders, and other hazardous diseases. Additionally, methanogens and other bacteria can convert inorganic mercury into a more toxic form, organic mercury, which mainly causes severe harm to human nerves and the brain^4–6^. Due to the risk of chronic poisoning, the Joint Food and Agriculture Organization and the World Health Organization (FAO/WHO) recommend that total mercury intake not exceed 5 μg/kg body weight per week, and the maximum permitted mercury concentration in drinking water is 30 nM^2,7,8^. Natural water bodies' contamination by Hg(II) mainly comes from dyes, batteries, mining, and pulp industries^9,10^.

In response to the problem of water contamination by the Hg(II) ions, many techniques have been employed to solve it^11^, such as bioremediation^12^, membrane filtration^13^, photocatalytic degradation^14^, electrochemical techniques^15^, ion exchange^16^, and adsorption^17^. Adsorption is seen to be a more desirable technique for toxic heavy metals removal from the contaminated water than the other mentioned methods due to its simplicity, high efficiency, cheapness, and ability to handle large-scale systems^18^. Consequently, effective adsorbent development is an essential issue in the adsorption process^19^. Many materials have been utilized as adsorbents like chitosan^20^, activated carbon^21^, clay^22^, cellulose-based materials^23^, nanosized metal oxides^24^, and metal–organic frameworks^25^.

Cellulose is a naturally occurring polymer that has various advantages as it is considered cost-effective, available, reusable, and environmentally friendly material. It can be derived from many natural sources, like cotton, bamboo, and straw. It has a relatively low adsorption efficiency because of its lack of functional groups. In order to increase the adsorption efficiency, the cellulose chemical modification is occurred by introducing new desired functional groups. Cellulose has an abundance of hydroxyl groups that can be chemically modified by esterification, grafting, oxidation, or etherification, in order to prepare highly efficient cellulose-based adsorbents^26–30^. When compared to unmodified cellulose, modified cellulose had higher efficiency for adsorbing heavy metal ions^31^. Cellulose that had been treated with a sulfo group had greater adsorption efficiency^32^.

Cellulose-based adsorbents have been reported for various contaminants remediation such as arsenic^33^, Cr(IV)^34^, (Hg(II), Cd(II), Cu(II), and Pb(II))^35^, uranium (VI)^36^, and Cu(II)^37^. Moreover, dialdehyde cellulose microfibers have been utilized for the removal of eriochrome black T dye^38^. The removal of Cu^2+^, Hg^2+^, and Pb^2+^ was investigated using guanyl thiosemicarbazide functionalized dialdehyde cellulose^39^. Recently, Akl et al. used a semicarbazide modified Flax fiber biomaterial for the Cr(VI) and Alizarin Red S anionic dye removal^40^.

Here, cellulose adsorption capability enhancement was obtained by the modification of it with cyanoacetohydrazide and CS_2_ in the presence of KOH in order to obtain adsorbent (DAC@CAH@SK_2_) rich with nitrogen and sulfur functional groups that acts as a chelating agent. Correspondingly, the effectiveness of the DAC@CAH@SK_2_ material in Hg^2+^ adsorption from aqueous solutions and various real water samples was investigated. Adsorbents modification by ligands containing sulfur, nitrogen, and oxygen functional groups have shown high binding affinity toward Hg(II) ions. Current research has focused on the development of adsorbents with sulfur/Nitrogen-based as these functional groups are well known for Hg(II) remediation efficiency enhancement through soft Lewis’s acid-based interaction. The multifunctional nature of DAC@CAH@SK_2_ is expected to enhance Hg(II) adsorption capacity^39–46^. As of now, very little work has been reported on the use of DAC@CAH@SK_2_ for the removal of Hg(II) ions from aquatic solutions.

According to our knowledge, cellulose modification using a nitrogen-containing ligand (cyanoacetohydrazide) and a Carbon disulfide has not been reported in the literature. Furthermore, no data were found on utilizing DAC@CAH@SK_2_ modified cellulose as an effective adsorbent/chelating agent for Hg^2+^ removal from actual polluted water samples.

Accordingly, the following goals guided the performance of the current study:

1. Design and synthesis of DAC@CAH@SK_2_ composite for adsorption of Hg^2+^ in aqueous solutions.
2. Characterization of the as-prepared DAC@CAH@SK_2_ composite utilizing physical (optical images), elemental analysis (CHNS), and spectroscopic (FTIR), SEM, ^1^HNMR and EDS, XRD, BET, and TGA instrumental performances.
3. Batch experiments of Hg^2+^ pollutant adsorption.
4. Investigating the optimum parameters required for optimal adsorption of the Hg^2+^, e.g., pH, temperature, the initial Hg^2+^ concentration, the mass of DAC@CAH@SK_2_ composite, and the oscillation time, ionic strength as well as the interfering ions.
5. Studying the various adsorption isotherm, kinetics, and thermodynamic parameters.
6. Hg^2+^ removal efficiency and reusability of DAC@CAH@SK_2_ composite comparison with other previously reported adsorbents.
7. Clarification of the mechanism involved in the process of Hg^2+^ adsorption onto DAC@CAH@SK_2_ composite.

## Experimental

### Materials

Cellulose powder, cyanoacetohydrazide, Carbon disulfide, hydrogen disulfide, potassium periodate (KIO_4_), potassium hydroxide (KOH), mercury chloride (HgCl_2_), HCl, NaOH, glacial acetic acid, ethanol, and NaCl were bought from Sigma Aldrich. A 4% potassium periodate solution was prepared by liquefy 4 g of KIO_4_ in 100 ml dist.H_2_O.

### Instrumentation

The pH_PZC_ of DAC@CAH@SK_2_ was estimated as follows: 100mg of the DAC@CAH@SK_2_ chelating agent was added to a 25 ml 0.01M NaCl pH-adjusted solutions (2–12) and then the mixtures were shaken at the equilibrated shaker for 48 h. NaCl pH adjustment occurred by 0.1 M of HCl and 0.1 M of NaOH. The final pH was recorded after the shaking and ΔpH was calculated as in the following Eq (ΔpH = pH_i_–pH_f_). In order to obtain the pH_PZC_ value, ΔpH was plotted against the initial pH (pH_i_). As the pH_PZC_ value, ΔpH = 0, is the point where X-axis (ΔpH) crosses the ΔpH vs pH_i_ curve^47,48^.

Fourier transform infrared, FTIR, spectra of DAC, DAC@CAH, DAC@CAH@SK_2_ composite, the DAC@CAH@SK_2_-Hg^2+^, as well as the native cellulose were obtained at wavenumber range (4000–400 cm^−1^) by a Perkin-Elmer, Spectrum RX I using KBr pellets. The DAC, DAC@CAH, DAC@CAH@SK_2_, and DAC@CAH@SK_2_-Hg^2+^surface morphologies were examined by scanning electron microscopy (A JSM-6510LV) that was used, also, to obtain the EDS (Energy Dispersive X-ray Spectroscopy) spectral investigation of the DAC@CAH@SK_2_-Hg^2+^.A PAN analytical X'Pert PRO diffractometer was employed for the X-ray diffraction (XRD) patterns of the DAC, DAC@CAH@SK_2_, and DAC@CAH@SK_2_-Hg^2+^samples investigation at (4–70°) range of 2-theta (2θ). The native cellulose, DAC@CAH, and DAC@CAH@SK_2_ materials specific surface area (S_BET_) was investigated utilizing the Brunauer Emmet Teller (BET) analysis (Size Analyzer (QUANTACHROME—NOVA 2000 Series). The CHNS composition determination of native cellulose, DAC@CAH, and DAC@CAH@SK_2_ was obtained by a Costech ECS-4010 elemental analyzer. The thermal stability of DAC@CAH@SK_2_ and DAC@CAH@SK_2_-Hg^2+^materials was examined by thermogravimetric analysis (Berkin Elmer TGA 4000) at a heating rate of 15 °C/min from 30 to 800 °C. The ^1^HNMR spectra of the prepared DAC and DAC@CAH@SK_2_ materials were measured in a mixture of DMSO/trifluoroacetic acid (TFA) using a Joel 500 MHZ Japan.

### Preparations

#### preparation of dialdehyde cellulose (DAC) and the aldehyde content (%) estimation

**Figure 1:**
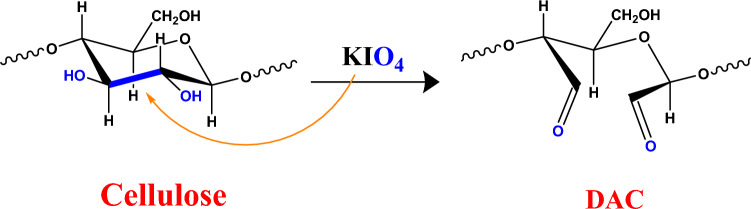
Preparation of 2,3 dialdehyde cellulose (DAC).

In the complete absence of light, the cellulose (one gram) oxidation occurred using 4% KIO_4_ (100 ml), as present in Fig. 1. The previous mixture was shaken for 6 h. The obtained oxidized cellulose (DAC) was washed by dist.H_2_O followed by the DAC drying step in an oven at 50 °C^39^. The aldehyde content (AC %) of the prepared DAC was determined as reported previously^39,40^, 0.1 g of the prepared DAC was added to 25 mL of 250 mM pH 4 adjusted hydroxylamine HCl then the mixture was stirred at room temperature in complete darkness for 2.5 h. Then, DAC was filtered and dried in an oven for 60 min at 70 °C. The filtrate was back-titrated utilizing 0.1 M NaOH to pH 4 and the reaction endpoint was achieved when the color shifted from red to yellow. The control experiment was performed by replacing the DAC with native material. The estimation of the prepared DAC aldehyde content percentage (AC (%)) has occurred using Eq. (1).

1 $${\text{AC }\%} = \frac{{{\text{NaOH Concentration }}\left( {{\text{V}}_{{{\text{sample}}}} - {\text{V}}_{{{\text{control}}}} } \right)}}{{{\raise0.7ex\hbox{${\text{m}}$} \!\mathord{\left/ {\vphantom {{\text{m}} {{\text{Mwt}}}}}\right.\kern-0pt} \!\lower0.7ex\hbox{${{\text{Mwt}}}$}}}}$$

Where V_sample_ and V_control_ are the volumes of NaOH in the case of oxidized cellulose and native cellulose powder, respectively. While m is sample weight and Mwt is cellulose molecular weight.

#### Preparation of cyanoacetohydrazide/carbondisulfide modified dialdehyde cellulose (DAC@CAH@SK_2_) sorbent

**Figure 2:**
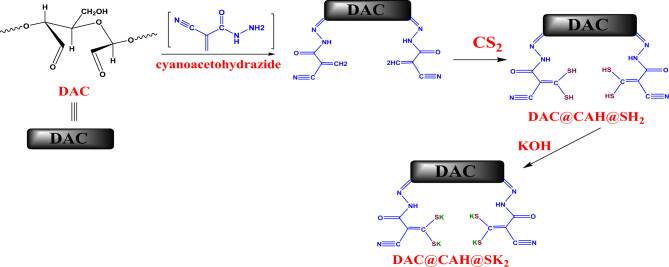
Synthesis of DAC@CAH@SK_2_.

At first, 4 g of cyanoacetohydrazide were dissolved in ethanol and were added to 1.5 g of DAC with the addition of 2 drops of glacial acetic acid. The previous mixture was allowed to reflux for 6 h at 70 °C to form DAC@CAH material. Then the mixture was filtered and washed with ethanol. After drying the DAC@CAH material at 45 °C, it was added to 10ml CS_2_ then 40 ml of ethanol was added. The mixture was stirred for 8 h at room temperature. Then 1ml of KOH solution was added to the previous mixture and allowed to stir for 15 min at room temperature. Finally, the mixture was filtered and the obtained DAC@CAH@SK_2_ material was washed with ethanol and dist.H_2_O and dried in an oven at 45^ο^C. The subsequent steps of preparation of DAC@CAH@SK_2_ are illustrated in Fig. 2.

### Adsorption and regeneration experiments

#### Batch adsorption

The adsorption behavior of Hg^2+^ using the DAC@CAH@SK_2_ material was investigated through batch adsorption experiments in 50ml stoppered bottles that contained 10 mL of Hg^2+^ solutions and DAC@CAH@SK_2_ dose. Various parameters were investigated including pH (from 1 to 10), metal ion concentration (25–400 mg/L), chelating agent weight (0.001–0.015g), and oscillation time (30–300 min). Unless specified otherwise, the pH, DAC@CAH@SK_2_ dose, concentration, and contact time were fixed to 6.0, 0.01g, 150 ppm, and 3.0 h, respectively. The pH was adjusted using diluted HCl and NaOH solutions. The concentration of Hg^2+^ upon each adsorption experiment was determined by ICP-OES (Agilent's 5100 instrument). The removal (%) and adsorption capacity at equilibrium (q_e_) was determined using Eqs. (2) and (3), respectively^47^.

2 $${\text{R }\%} = \frac{{{\text{C}}_{{\text{i}}} - {\text{C}}_{{\text{f}}} }}{{{\text{C}}_{{\text{i}}} }} \times {1}00$$

3 $${\text{q}}_{{\text{e}}} = \frac{{\left( {{\text{C}}_{{\text{i}}} - {\text{C}}_{{\text{f}}} } \right){\text{*v}}}}{m}$$

C_i_ and C_f_ are the initial and equilibrium Hg^2+^ concentration (ppm), respectively. While m (g) is the DAC@CAH@SK_2_ dose and V (L) is the adsorbate solution volume.

#### Desorption and regeneration

Regeneration of DAC@CAH@SK_2_ composite was obtained through 5 adsorption–desorption following cycles. Hg(II) adsorption experiments were carried out by utilizing 0.01 g of DAC@CAH@SK_2_ and 10 mL (150 mg/L) of Hg(II) solution at pH 7 for 2 h. For desorption investigation, 10 ml of HNO_3_ 0.1M- thiourea 0.1M mixture (1:1) and 0.01 g of DAC@CAH@SK_2_-Hg^2+^ were shaken for 2 h^49^. Finally, the regenerated DAC@CAH@SK_2_ chelating agent was reused for another five repeated adsorption–desorption cycles.

#### Adsorption Selectivity

A total of 10 mg of DAC@CAH@SK_2_ composite was added to a 10 ml solution of binary metal ions and multiple metal ions synthetic mixtures (with the same concentrations of each metal ion (150 mg L^−1^)) and shaken at 25 °C for 2 h. The added metal ions concentrations were estimated by ICP OES and the adsorption capacities were calculated with Eq. (3), and then the adsorption selective coefficient (α) was defined as presented in Eq. (4):

4 $${\upalpha } = \frac{{{\text{The adsorption capacity of Hg }}\left( {{\text{II}}} \right){\text{ on DAC}}@{\text{CAH}}@{\text{SK}}2{ }}}{{{\text{The adsorption capacity of coexisting metal ion on DAC}}@{\text{CAH}}@{\text{SK}}2{ }}}$$

### Application

150 mg/L of Hg^2+^ was spiked to the real (sea, waste, and tap) water samples. Before the spiking step, the real water samples were digested by adding a mix of K_2_S_2_O_8_ (0.5 g) and H_2_SO_4_ (5ml, 98% (w/w)) to 1000 ml of each water sample and then heated for 120 min at 90 °C for complete digestion of presented organic materials. After cooling to room temperature, 0.01 g of DAC@CAH@SK_2_ was added to the prepared samples, and the pH value was adjusted to 7 with continuous shaking for 180 min. The solutions were centrifuged and again another 0.01 g of DAC@CAH@SK_2_ was added to the supernatant to ensure the complete separation of analytes. The remaining Hg^2+^ was determined using ICP OES.

## Results and discussion

### Materials' design

#### DAC synthesis

The KIO_4_ oxidizing agent is a known selective one that is utilized for the oxidation of two OH groups that are present on the glucopyranoside ring's C_2_–C_3_ neighboring carbon atoms. KIO_4_ cleaves the bond between C_2_–C_3_ and the OH groups are converted into two dialdehyde groups. The oxidation degree which represents the percentage of monosaccharide units that reacted with KIO_4_ is calculated by aldehyde content determination^39^. The AC % of the synthesized DAC is 38.93% as shown in Table 1.

**Table 1:** Volumetric titration of oxidized dialdehyde-cellulose (DAC) for average aldehyde content percentage AC% estimation.

V_control_ (ml)	V_sample_ (ml)	C_NaOH_ (M)	m (gm)	AC %	Average AC%
0	2.42	0.1	0.1	38.72	38.93
0	2.45	0.1	0.1	39.2	
0	2.43	0.1	0.1	38.88	

#### Physicochemical properties of native cellulose, DAC@CAH, and DAC@CAH@SK_2_ composite

Specific surface area is an essential parameter, as it has a great effect on adsorbent capacity toward pollutants. The surface area of DAC, DAC@CAH, and DAC@CAH@SK_2_ was measured and is presented in Table 2**.** The surface area was decreased by modification of cellulose with cyanoacetohydrazide to 8.137 m^2^/g. This decrease may be due to the reaction of cyanoacetohydrazide with the cellulose. While the surface area increased after the reaction with CS_2_ to 268.729 m^2^/g, which may be returned to the grafting of SH groups into the material.

The solubility of DAC@CAH@SK_2_ was studied by utilizing various solvents like sodium hydroxide (0.1–1 M), HCl (0.1–1 M), and ethanol 99.9%. It was noticed that DAC@CAH@SK_2_ is not soluble in any of the utilized solvents.

**Table 2:** Surface area of the native cellulose and the prepared materials**.**

Sample	Surface area, m^2^ g^−1^
Native cellulose	10.4
DAC@CAH	8.137
DAC@CAH@SK_2_	268.729

### Characterization

#### Elemental analysis

Table 3 illustrates the EA results of native cellulose, DAC@CAH, and DAC@CAH@SK_2_ composite. The results obtained indicate that the nitrogen content significantly increased to 11.73% after the modification that occurred through the cellulose oxidation followed by condensation with cyanoacto hydrazide. Nitrogen content decreased to 8.43% with increasing sulfur content to 12.86% after the reaction with Carbon disulfide. These results confirm that DAC-CAH and DAC@CAH@SK_2_ are successfully formed. The concentration of the inserted cyanoaceto-hydrazide units was calculated to be approximately 1.557 mmol g^−1^.

**Table 3:** Elemental analysis of native cellulose and the prepared compounds.

Material	C%	H%	N%	S%
Native cellulose	44.5	7.14	0	0
DAC@CAH	46.93	5.63	11.73	0
DAC@CAH@SK_2_	33.72	3.64	8.43	12.86

#### Scanning electron microscope (SEM)

**Figure 3:**
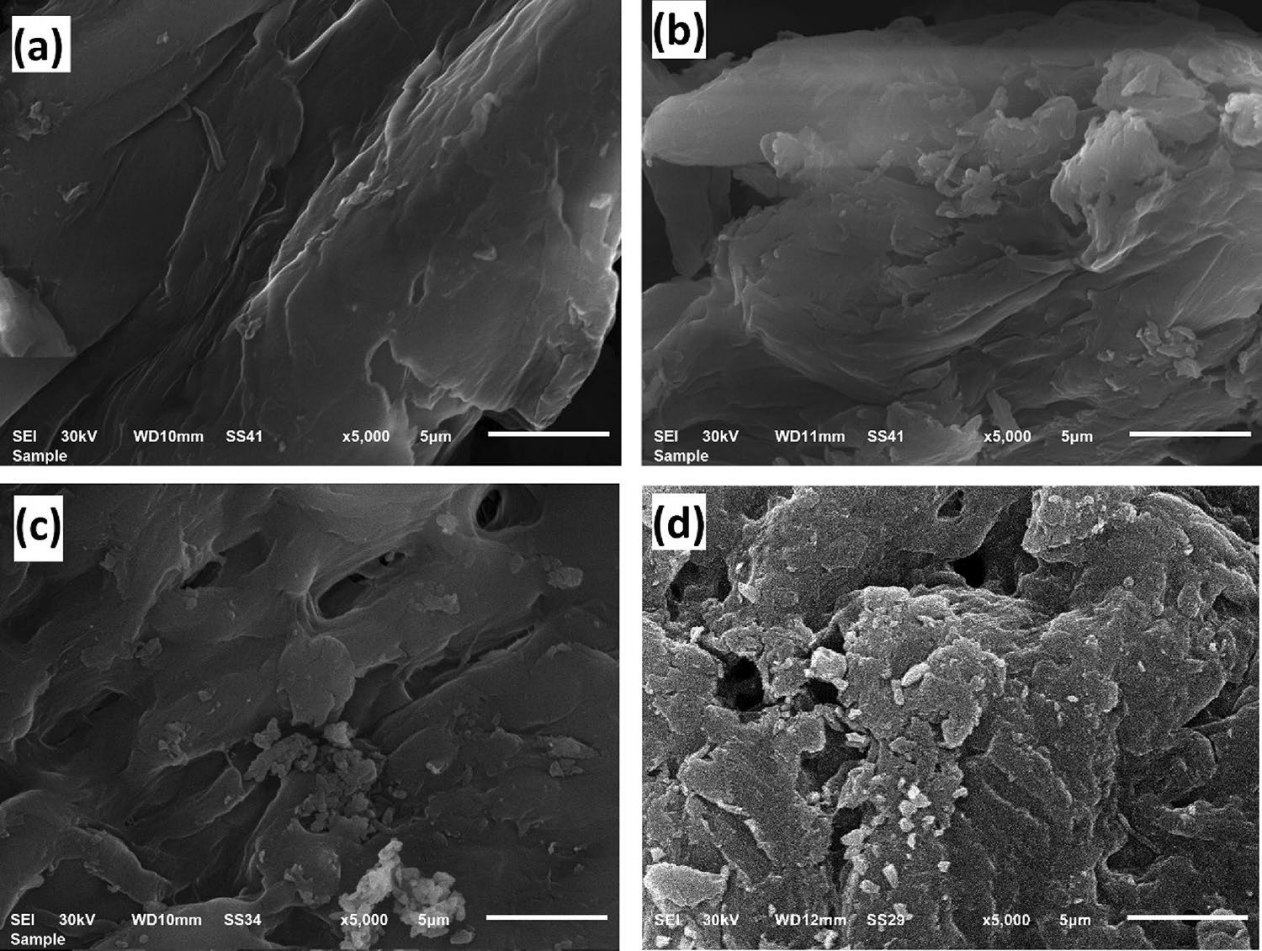
(**a**) Oxidized cellulose (DAC), (**b**) DAC@CAH, (**c**) DAC@CAH@SK_2_, and (**d**) DAC@CAH@SK_2_-Hg^2+^.

Surface morphologies of DAC, DAC@CAH, DAC@CAH@SK_2_, and DAC@CAH@SK_2_-Hg^2+^are shown in Fig. 3a–d, respectively. Figure 3a and b showed that DAC@CAH has more roughness than DAC material which may be attributed to the chemical modification by cyano-aceto hydrazide. DAC@CAH@SK_2_ has more roughness and porous surface than DAC@CAH as shown in Fig. 3c, which may be attributed to the modification of DAC@CAH with CS_2_ in the presence of KOH. As shown in Fig. 3d, the SEM of DAC@CAH@SK_2_-Hg^2+^ shows a brighter surface than the other materials because mercury has better electric conductivity than the modified cellulose materials^50,51^.

#### FTIR spectra

**Figure 4:**
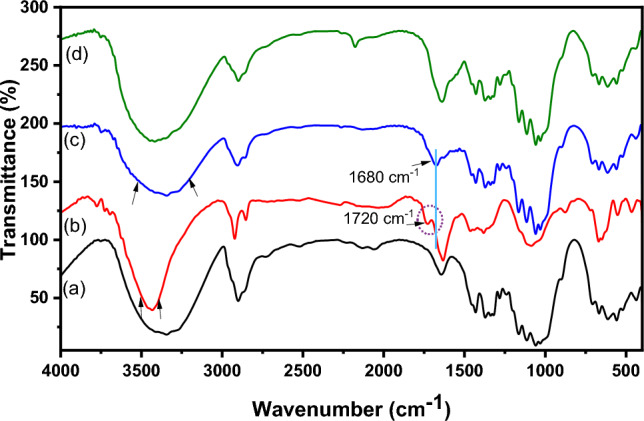
FTIR spectra of (**a**) native cellulose, (**b**) DAC, (**c**) DAC@CAH, and (**d**) DAC@CAH@SK_2_.

**Figure 5:**
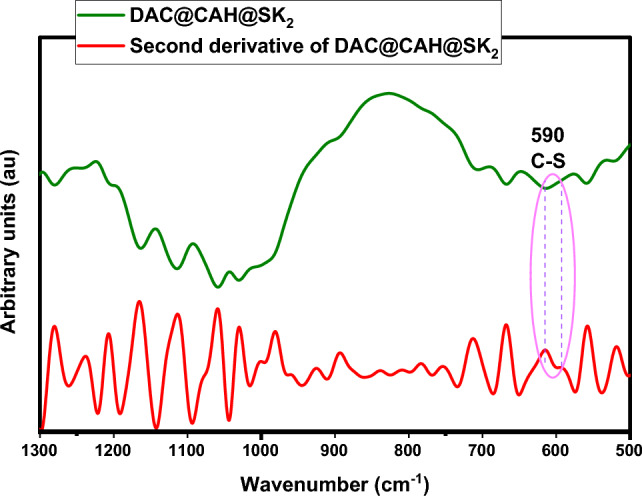
Second derivative spectra of DAC@CAH@SK_2_.

FTIR spectra of native cellulose, its derivatives (DAC, DAC@CAH, and DAC@CAH@SK_2_ composite), and DAC@CAH@SK_2_-Hg^2+^ are represented in Fig. 4. Native cellulose (Fig. 4a) showed a number of distinctive peaks as the C–O stretching vibrations that appeared in the range of 1000–1200 cm^−1^. While those in the range of 1260–1410 cm^–1^ and 3200–3600 cm^−1^ are assigned to OH bending and stretching vibrations, respectively. Moreover, the peaks of C–H stretching vibrations that are present between 2700 and 3000 cm^−1^^39^. FT-IR spectrum of DAC is present in Fig. 4b and exhibits a new peak at about 1730 cm^−1^ which may be assigned to the C=O stretching vibrations of the aldehyde group (RCHO)^40^. DAC Modification with Cyanoactohydrazide results in some shifts in the IR spectrum of the DAC@CAH, an observed peak at about 1680 cm^−1^, which may be returned to C=N formation between the DAC' aldehyde groups present and amino groups of the added Cyanoactohydrazide as present in Fig. 4c and^23^. Moreover, the peak between 3600 and 3100 cm^−1^ became broader due to the OH and amino groups' absorption peaks overlapping^52^. The spectrum of the DAC@CAH@SK_2_ composite, Fig. 4d**,** shows the presence of cyano group stretching vibrations at about 2174 cm^-1^ that may be appeared after the reaction with CS_2_ because of the tautomerism phenomenon^53^. Furthermore, due to the presence of overlapping bands at the range of 500–600 cm^−1^, the C–S peak was not visible individually^54^. The second derivative FT-IR was obtained in the 1300–500 cm^−1^ range to confirm the presence of C–S characteristic peak. As presented in Fig. 5 for DAC@CAH@SK_2_ through the 2nd derivative, a band at 590 cm^−1^ attributed to C–S stretching vibrations was detected^55,56^.

#### ^1^H-NMR

**Figure 6:**
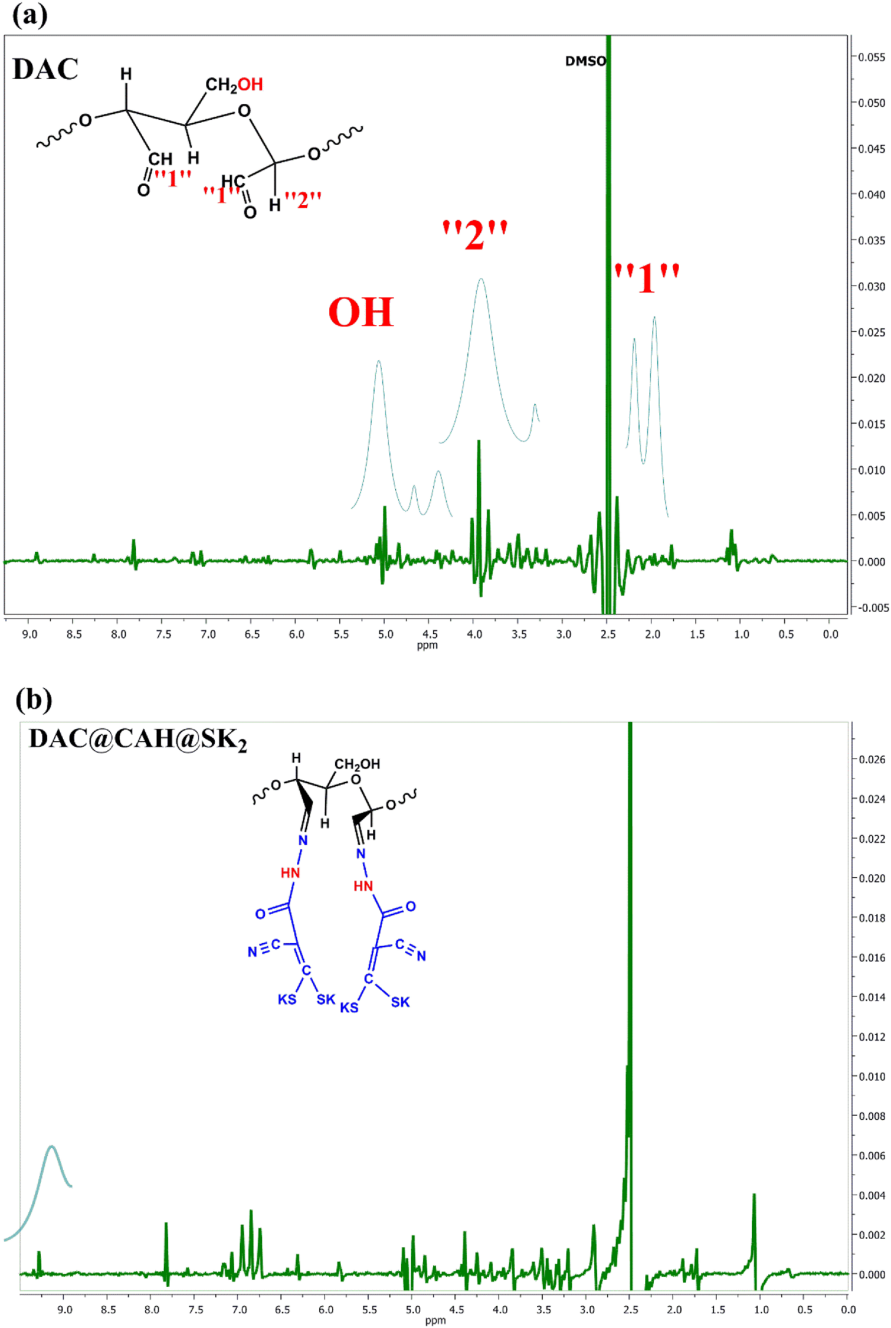
^1^H NMR of (**a**) DAC and (**b**) DAC@CAH@SK_2_.

Besides the XRD technique, both liquid and solid phases of NMR are used to study the cellulose structure. Also, the solid phase of ^13^C NMR is used to investigate the various cellulose polymorphs but it is less available and requires more extensive investigations than liquid phase NMR^40,57,58^. In all molecular solvents, both DAC and DAC@CAH@SK_2_ cellulosic materials are completely insoluble. The ^1^H NMR of DAC and DAC@CAH@SK_2_ were investigated by utilizing the DMSO/Trifluoroacetic acid mixture^58^. Figure 6 represents the ^1^H NMR of DAC and DAC@CAH@SK_2_. The ^1^H NMR of DAC present in Fig. 6a showed a peak at 2.03 ppm related to the proton that is present on C_2_ or C_3_ of the DAC. Broad peaks that appeared at 4 ppm and 4.96 ppm are related to C_1_ proton and OH, respectively. Figure 6b represents the ^1^H NMR of DAC@CAH@SK_2_ that showed a new prominent peak, broad signal near 9.28 ppm that could be is related to protons of NH of the cyanoacetohydrazide^40,59^.

#### Thermal analysis (TGA)

**Figure 7:**
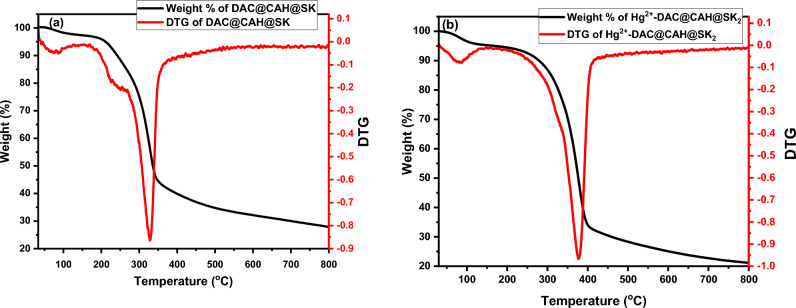
Thermal analysis of (**a**) DAC@CAH@SK_2_ and (**b**) DAC@CAH@SK_2-_Hg^2+^.

Thermogravimetric analyses for the DAC@CAH@SK_2_ before and after mercury ions adsorption were carried out in the temperature range of 30–900 °C to give information about materials thermal stability, Fig. 7 The thermograms demonstrated that each compound undergoes a series of different degradation steps. The thermal degradation of DAC@CAH@SK_2_ and DAC@CAH@SK_2_-Hg^2+^ were studied under the same conditions and the total residues were determined as following 27.89% and 21.15%, respectively. The lower value of total residues of DAC@CAH@SK_2_-Hg^2+^compared to the parent one (DAC@CAH@SK_2_) suggests that mercury ions show a noncatalytic degradation effect during their complexation with the modified cellulose material^60^.

#### X-ray diffraction

**Figure 8:**
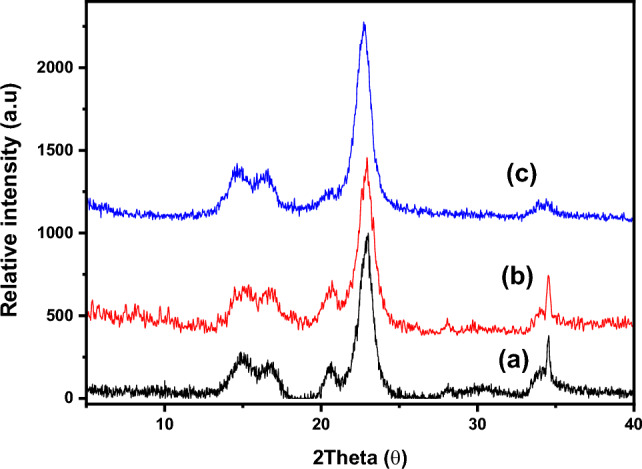
XRD patterns of (**a**) Cellulose, (**b**) DAC, and (**c**) DAC@CAH@SK_2_.

The diffractograms of the untreated cellulose, DAC, and DAC@CAH@SK_2_ are presented in Fig. 8. Key diffraction peaks of the cellulose were shown in the XRD patterns at roughly 15.178º, 16.59º, 20.5º, 22.835º, 28.05º, and 34.475º, with major intensities at peak values of 15.178º, 16.59º, 22.835º, and 34.475º. The primary peaks associated with crystallographic planes were (11̅0), (110), (200), and (004), respectively and these peaks were discovered to be related to cellulose type I^61–63^. The positions of these investigated peaks stay similar in DAC and DAC@CAH@SK_2_ materials with some slight shifting in addition to the samples' crystallinity indices (CrI) were calculated according to the Segal method (Eq. 5) and found to be 73.19, 71.77, and 69.04% for untreated cellulose, DAC, DAC@CAH@SK_2_. This may indicate that the crystallinity of the functionalized samples (DAC and DAC@CAH@SK_2_) was slightly affected by the adopted chemical modification^40,64^. The crystallinity decrease is returned to the glucopyranose ring opening and cellulose backbone destruction that result from cellulose oxidation by the KPI and 2,3 dialdehyde cellulose (DAC) formation^65,66^. The (CrI) value of DAC@CAH@SK_2_ is lower than that of DAC which indicates that the DAC@CAH@SK_2_ is more amorphous than DAC. This could be illustrated according to their chemical structure as the DAC@CAH@SK_2_ has a more compact structure than the DAC^40,64^.

5 $$\text{CrI}\% = \frac{{I_{200} - I_{am} }}{{I_{200} }} \times 100$$

As I_200_ and I_am_ are the maximum intensity of 200 diffraction plane at 2θ = 22.835° and the diffraction intensity of the amorphous phase at 2θ of 15.178°, respectively.

### Adsorption studies

#### Point of zero charge (pH_PZC_)

**Figure 9:**
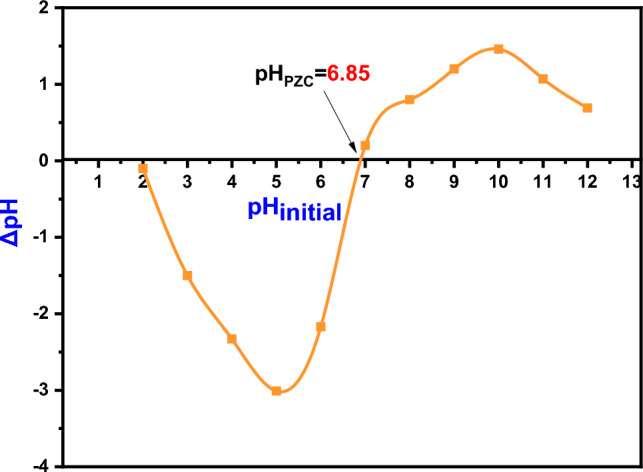
pH_PZC_ of the DAC@CAH@SK_2_.

In order to understand the Hg(II) adsorption mechanism by the prepared composite, the point of zero charge of DAC@CAH@SK_2_ composite was studied as presented in Fig. 9. This investigation was obtained by measuring the pH at the point of zero charge (pH_PZC_). Commonly, the chelating agent will show greater affinities for cations at a pH value higher than the value of its pH_PZC_ and vice versa. The pH_PZC_ value obtained for the DAC@CAH@SK_2_ composite was approximately 6.85. Hg(II) adsorption by the DAC@CAH@SK_2_ was expected to be enhanced at a pH value higher than the pH_PZC_ value.

#### Effect of pH

**Figure 10:**
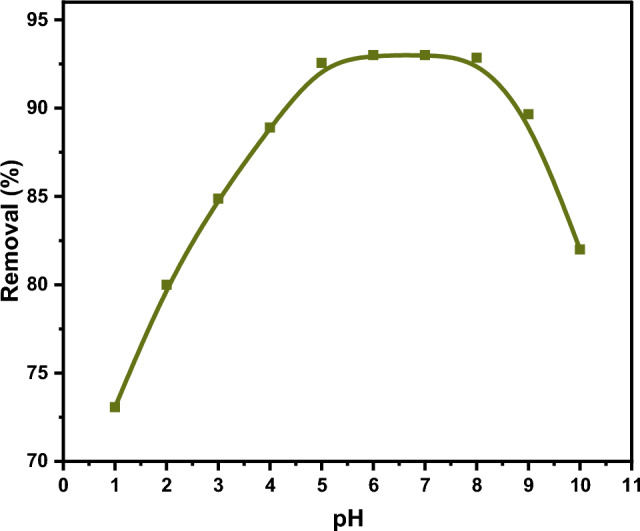
pH effect on Hg^2+^ adsorption onto DAC@CAH@SK_2_.

The pH is considered an essential parameter as it can influence the solubility and the ionization degree of the studied chelating agent. Also, it can affect metal ion speciation. The adsorption behavior of DAC@CAH@SK_2_ chelating agent has been investigated at the pH range of 1–10, which was selected to avoid metal precipitation that occurs in the very alkaline medium. The experiment was studied using 0.01 g of DAC@CAH@SK_2_ composite immersed in 10 mL of 150 ppm Hg(II) solution for 180 min. As shown in Fig. 10, the Hg^2+^ adsorption increased with pH increasing from 1 to 5 and became constant at pH range 5–8 then it decreased at pH higher than 8.

The prevailing species for the divalent mercury At pH 2 are HgCl_2_, HgCl^+^, Hg^2+^, and HgOHCl by percent % 63.25, 25.20, 3.9, and 2, respectively; in addition to the presence of HgOH^+^ and Hg(OH)_2_ in very minor quantities. While the prevailing species at pH 4 are 39.90% Hg(OH)_2_, 25.20% HgOHCl, and 10.02% HgCl_2_ besides the other species that are present in very small percent such as HgOH^+^, HgCl^+^, and Hg^2+^. The Hg(OH)_2_ and HgOHCl species are the prevalent ones at the pH range from 6 to 8 and present by percent 79.62% and 10.02%, respectively^67–69^. The higher affinity of the DAC@CAH@SK_2_ composite to Hg(II) at higher pH is that the prepared composite contains SK groups that consider soft bases while Hg(II) ions are soft acids. In accordance with the theory of Pearson, throughout an acid–base reaction hard acid coordinates with the hard base while soft acid coordinates with the soft base. Neutral molecules consider softer acids.

Our experimental results indicate that the pH is a major parameter in Hg(II) adsorption process by the DAC@CAH@SK_2_ composite. The Hg(II) adsorption to cellulose-based adsorbents in response to pH changes has been investigated by several researchers. Bisla et al.^70^ investigated the Hg(II) adsorption by l-methionine-functionalized cellulose nanofibers adsorbent Which has pHpzc equals 7.8 in the pH range (2–10) and declared the very low Hg(II) adsorption capacity at pH value 2, then a constant adsorption capacity increase in the range of pH 4–8, and then followed by a slight decrease at pH value 10. Arias et al.^71^ reported that the Hg(II) removal % by lignocellulosic materials was very low at pH 2 and then increased relatively up to pH 9. Anirudhan and Shainy^67^ investigated the divalent Hg ions adsorption into 2-mercapto benzamide-modified nano cellulose composite with a pH_pzc_ value equal to 6.5 in the pH range of 3–11. They indicated that the divalent mercury adsorption increased by pH value increasing from 3 to 8 and then decreased sharply as the pH value increased to 11. These investigations recommend that Hg(II) adsorption to cellulose-based adsorbents relies on the utilized adsorbent/chelating agent properties such as pH_pzc_, functional groups, etc. in addition to the speciation of the divalent mercury in the studied solution.

#### Effect of chelating agent dose

**Figure 11:**
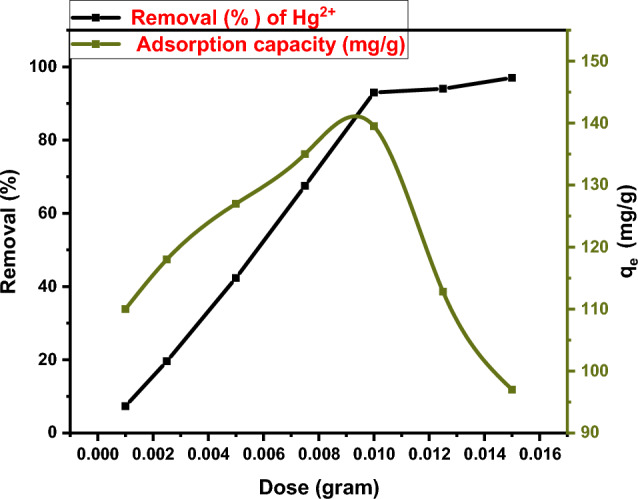
Effect of sorbent dose on adsorption of Hg^2+^ (conditions: 10 ml aqueous solution of 150 mg/L for Hg^2+^ solution for 3 h at pH 6).

The influence of DAC@CAH@SK_2_ composite dose on Hg^2+^ adsorption capacity (mg/g) and removal efficiency (%) was obtained by utilizing various doses of it and the results are present in Fig. 11. The Hg^2+^ removal % increased rapidly as the chelating agent dosage increases from 0.001 to 0.01 g, with an increase in the adsorption capacity from 110 to 139.5 mg/g. While the removal % slightly increased from 93 to 97% by increasing the DAC@CAH@SK_2_ composite dose from 0.01 to 0.015 g this was accompanied by a decrease in adsorption capacity from 139.5 to 97 mg/g. This may be returned to the increase in DAC@CAH@SK_2_ dose results in the specific surface area increase which means more available adsorption sites.

#### Effect of initial concentration of Hg^2+^

**Figure 12:**
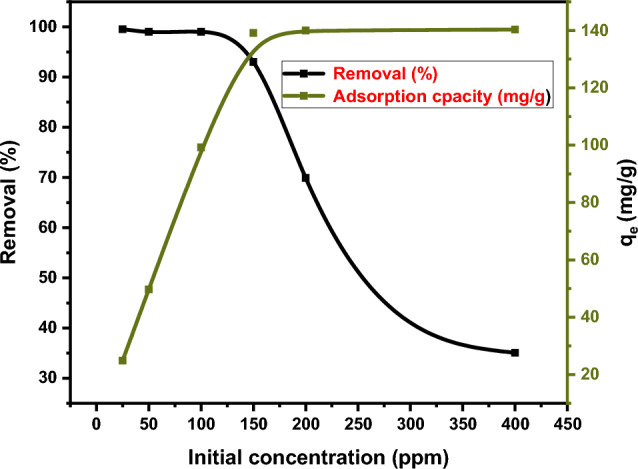
Effect of Hg^2+^ initial concentration (conditions: 0.01g of DAC@CAH@SK_2_ was taken at pH 6 for 3 h in range 25 ppm-400 ppm of Hg^2+^).

To investigate the effect of initial concentration on Hg^2+^ adsorption capacity, a 10 ml solution of Hg^2+^ at a fixed-dose of DAC@CAH@SK_2_ chelating agent 0.01 g for Hg^2+^ was taken at pH 6 for 3 h in range (25–400 ppm). After that, initial concentrations were varied and the corresponding capacities and removal percentages were obtained as shown in Fig. 12. It was noticed that the DAC@CAH@SK_2_ adsorption capacity for Hg^2+^ increased from 24.88 to 139.1 mg/g with Hg^2+^ initial concentration increasing from 25 to 150 ppm. Moreover, with the increase of Hg^2+^ initial concentration from 150 to 400 ppm, the DAC@CAH@SK_2_ composite tends to stabilize.

#### Adsorption isotherms

The adsorption isotherm can be defined as follows; the studied adsorbate concentration relation with the adsorbed pollutant amount (q_e_) at the adsorbent (chelating agent) surface at a fixed temperature. Dubinin–Radushkevich (D-R), Langmuir, and Freundlich's isothermal models, which can indicate the maximum adsorption capacity (q_e_) and binding affinity, were applied in the linear form and the parameters were determined as present in Eqs. (6), (7) and (8), respectively. The dimensionless equilibrium factor (R_L_) presented in Eq. (9), is an important parameter that is used in adsorbent-sorbate affinity prediction. Its values are explained as follows: if the R_L_ value is found to be greater than 1.0 this means that the investigated material is unsuitable and unfavorable, while if it is found to be (0 < R_L_ < 1), (R_L_ = 0), or (R_L_ = 1) this means the reaction is favorable, irreversible, or linear, respectively^23,35^. The Dubinin–Radushkevich model studies adsorption energetically and creates the assumption that the adsorption process relates to pore volume and surface porosity. Equation (10) represents the E_DR_ which can be defined as following the adsorption mean free energy obtained from the D–R model. It reveals whether the adsorption process is chemical (8 < EDR < 16 kJ mol^−1^) or physical (E_DR_ is lower than 8 kJ mol^−1^)^40^.

6 $$\ln \text{q}_{e} = \ln \text{q}_{m} - \text{k}\upvarepsilon^{2}$$

7 $$\frac{{{\text{C}}_{{\text{e}}} }}{{{\text{q}}_{{\text{e}}} }} = \frac{1}{{{\text{k}}_{{\text{L}}} {\text{q}}_{{\text{m}}} }} + \frac{{{\text{C}}_{{\text{e}}} }}{{{\text{q}}_{{\text{m}}} }}$$

8 $$\ln q_{e} = lnK_{f} + \frac{1}{n}lnC_{e}$$

9 $$R_{l } = \frac{1}{{1 + K_{l} C_{ \circ } }}$$

10 $$E_{DR} = \frac{1}{{\sqrt {2K} }}$$

where C_e_ (mg/L), 1/n, K_L_ (L/mg), K_F_ (mg/g), and K are the Hg^2+^ concentration at equilibrium, the heterogeneity factor, Langmuir, Freundlich, and the Dubinin–Radushkevich constants, respectively. While, q_e_ and q_m_, which are expressed in mg/g, are the Hg^2+^ capacity at equilibrium and adsorption maximum amount. R which its value equals 8.314 J/mol and T which is expressed in Kelvin are the gas constant and the temperature, respectively. ε is the adsorption potential and is presented in Eq. (11).

11 $${\upvarepsilon } = {\text{RTln}}\left( {1 + \frac{1}{{{\text{C}}_{{\text{e}}} }}} \right)$$

**Figure 13:**
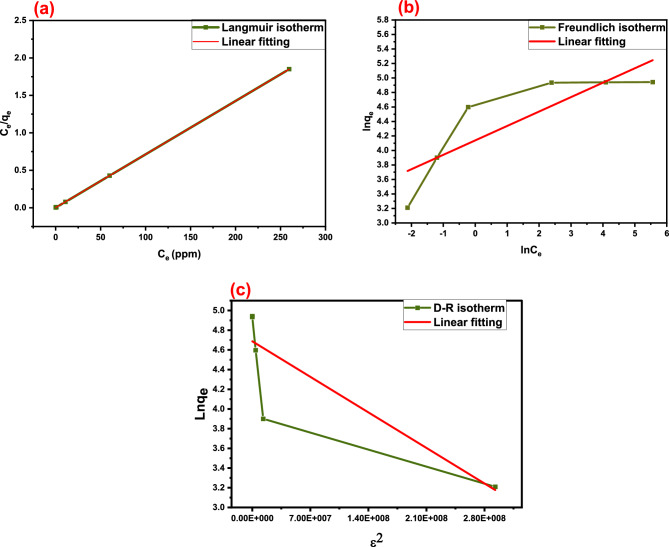
Adsorption isotherms for Hg(II) adsorption by DAC@CAH@SK_2_: (**a**) Langmuir isotherm model, (**b**) Freundlich isotherm model, and (**c**) D-R isotherm model.

The Langmuir, Freundlich, and D–R isotherms determined for the Hg(II) adsorption utilizing DAC@CAH@SK_2_ chelating agent are presented in Fig. 13 and their derived parameters (K_L_, K_f_, K, n, and q_m_) are given in Table 4. The Hg(II) adsorption process using DAC@CAH@SK_2_ composite follows the Langmuir isotherm model as the R^2^ value for the Langmuir isotherm model is higher than that of Freundlich as presented in Table 4. This explains that the binding of Hg(II) ions to the active sites of the DAC@CAH@SK_2_ composite is a chemisorption process (mono-layer). The R_L_ value was calculated and found to lie between 0 and 1 as shown in Table 4 which implies that the Hg(II) adsorption by DAC@CAH@SK_2_ composite is a favorable process that proves the applicability of the prepared composite for Hg(II) remediation from solutions^40^. The current adsorption process' E_DR_ was calculated from the D-R model as presented in Eq. (10) and found to be 9.847 lies in the range of 8–16 kJ mol^−1^ which reveals that the Hg(II) adsorption process onto the DAC@CAH@SK_2_ composite was chemisorption^40^.

**Table 4:** Adsorption isotherm parameters of Hg(II) by DAC@CAH@SK_2_.

Material	Langmuir isotherm constants			
	K_L_ (L/g)	q_m_ (mg/g)	R^2^	R_L_
DAC@CAH@SK_2_-Hg^2+^	2.777	140.65	1	0.0024

Material	Freundlich isotherm constants			
	K_F_	n		R^2^
DAC@CAH@SK_2_-Hg^2+^	1.22	0.2416		0.72685

Material	D–R isotherm constants			
	K	E (kJ/mole)	q_m_ (mg/g)	R^2^
DAC@CAH@SK_2_-Hg^2+^	5.156 × 10^–9^	9.847	108.58	0.72272

#### Effect of oscillation time and adsorption kinetics

**Figure 14:**
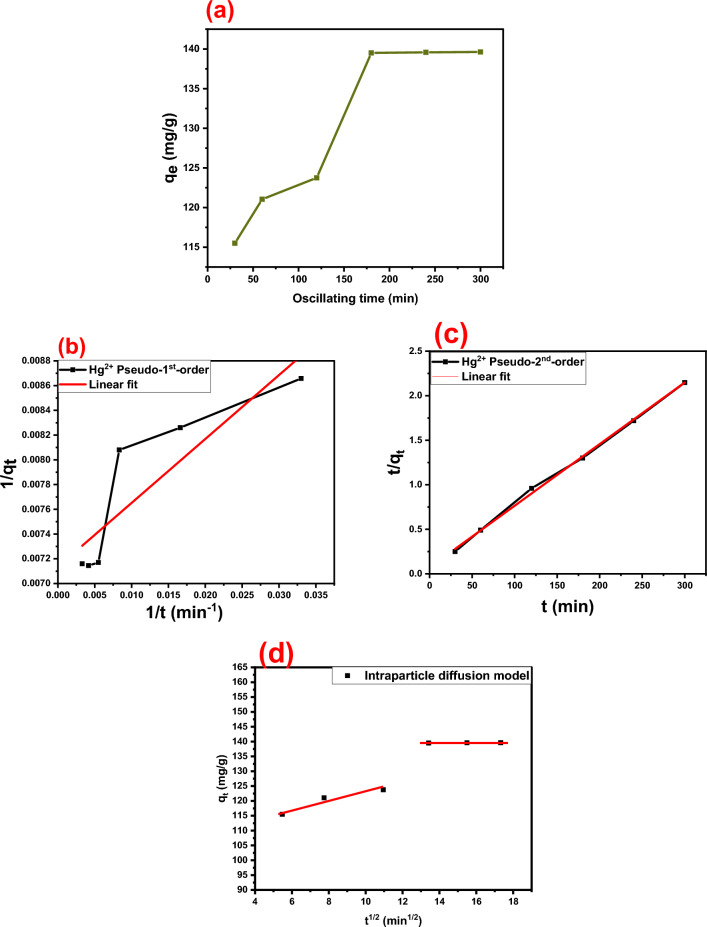
(**a**) Effect of oscillation time on adsorption of Hg^2+^ (conditions: 0.01 g of DAC@CAH@SK_2_, 10 ml of 150 ppm Hg^2+^, Temp.: 25 °C, time: 30–300 min), (**b**) Pseodo-1st-order kinetic model for Hg^2+^ adsorption, (**c**) Pseodo-2nd-order kinetic model for Hg^2+^ adsorption, and (**d**) IPD kinetic model for Hg^2+^ adsorption.

In order to investigate the Hg(II) adsorption mechanism using the DAC@CAH@SK_2_ material, the kinetic studies have occurred by examining the influence of oscillating time at various times from 30 to 300 min using 0.01 g of DAC@CAH@SK_2_ which was added to a series of glass bottles contain 10 ml of 150 ppm Hg(II). Figure 14a illustrates the adsorption capacity of DAC@CAH@SK_2_ at different oscillating times. It is noticed that the adsorption capacity of DAC@CAH@SK_2_ increased with the increase of oscillating time from 30 to 180 min to reach q_e_ value 139.5 mg/g. At an oscillating time of 180 min, the DAC@CAH@SK_2_ adsorption capacity became constant and the Hg(II) adsorp­tion attained equilibrium.

In order to determine the Hg^2+^ adsorption rate-limiting step, kinetic investigations were carried out using three typical models; pseudo-1st-order, pseudo-2nd-order and the intraparticle diffusion model (IPD) model which are shown in Eqs. (12), (13) and (14), respectively.

12 $$\frac{1}{{{\text{q}}_{{\text{t(ads)}}} }} = \frac{{{\text{k}}_{1} }}{{{\text{q}}_{{\text{e(ads)}}} }}{\text{t}} + \frac{1}{{{\text{q}}_{{\text{e(ads)}}} }}$$

13 $$\frac{{\text{t}}}{{{\text{q}}_{{\text{t(ads)}}} }} = \frac{1}{{{\text{k}}_{2} {\text{q}}_{{\text{e(ads)}}}^{2} }}{\text{t}} + \frac{1}{{{\text{q}}_{{\text{e(ads)}}} }}{\text{ t}}$$

14 $$q_{t} = k_{diff} *t^{0.5} + C$$

The adsorption efficiency for Hg^2+^ at equilibrium and at a certain time t (min) are expressed as q_e_ (mg/g) and q_t_ (mg/g), respectively. As well as K_1_, K_2_, and K_diff_ are pseudo-1st-order, pseudo-2nd-order, and IPD constants, respectively. The C which is defined as the IPD equation intercept, was utilized in order to investigate the impact of the boundary layer. It was discovered that as the intercept value increased, the contribution of the rate-limiting step got higher.

Figure 14b–d presents the experimental data fitting to Pseudo-1st-order (PFO), Pseudo-2nd-order (PSO), and Intraparticle-diffusion (IPD) kinetic models**.** Moreover, the kinetic parameters (K_1_, K_2_, K_diff_, q_e_1ads, q_e_^2^ads, and R^2^) derived from the three models are shown in Table 5. The adsorption of Hg(II) by DAC@CAH@SK_2_ achieved equilibrium within 3 h (Fig. 14a). When the correlation coefficients, R^2^, of both PFO and PSO models were compared, it was discovered that the results fit the PSO kinetic model better. It was noticed that two-line components appeared rather than a single one passing through the origin in the IPD model graph (Fig. 14d), which indicates that Hg^2+^ adsorption onto DAC@CAH@SK_2_ composite includes different diffusion stages that take place on and inside the DAC@CAH@SK_2_ surface. In this case, it was demonstrated that it is not possible to describe the adsorption with one kinetic model. The IPD model for Hg^2+^ adsorption by the DAC@CAH@SK_2_ composite indicates that the adsorption provides various diffusion stages that occurred on the DAC@CAH@SK_2_ composite surface and inside its surface. In the beginning, numerous active sites were available so the adsorption occurred quickly. Then, with oscillating time passing the DAC@CAH@SK_2_ composite active sites decreased and the diffusion of Hg^2+^ into pores becomes more difficult so the adsorption becomes slower^40,72,73^.

**Table 5:** Kinetic parameters for the adsorption of Hg^2+^ by DAC@CAH@SK_2_**.**

Material	First-order model		
	K_1_ (min^−1^)	q_e1ads_ (mg/g)	R^2^
DAC@CAH@SK_2_-Hg^2+^	7.25	141	0.77949

Material	Second-order model		
	k_2_ (g/(mg min))	q_e2ads_ (mg/g)	R^2^
DAC@CAH@SK_2_-Hg^2+^	5.5677*10^–4^	146.19	0.9977

Material	Intraparticle diffusion model		
	K_diff_		R^2^
DAC@CAH@SK_2_-Hg^2+^	0.388667		0.8961

#### Thermodynamics

**Figure 15:**
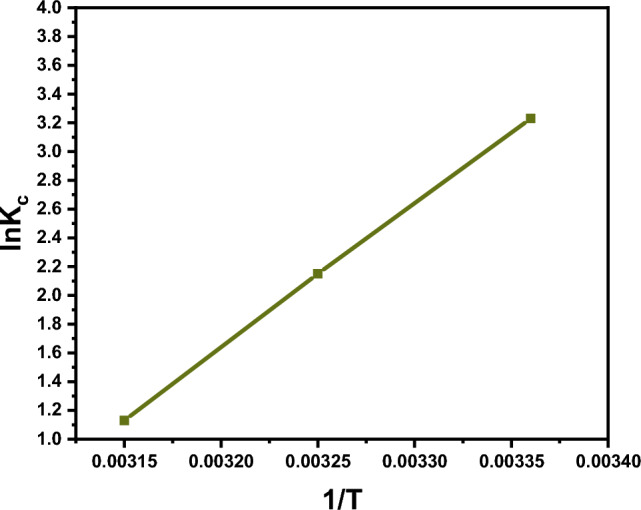
Plot of ln K_C_ vs (1/T) absolute temperature for the adsorption of Hg^2+^

To study the nature of the Hg^2+^ adsorption process onto the DAC@CAH@SK_2_ surface in terms of spontaneity and feasibility and to estimate the degree of randomness at the solid/liquid interface, adsorption thermodynamic parameters ($\Delta {\text{G}}_{{{\text{ads}}}}^{{\text{o}}}$, $\Delta {\text{H}}_{{{\text{ads}}}}^{{\text{o}}}$, and ($\Delta {\text{S}}_{{{\text{ads}}}}^{{\text{o}}}$)) were determined at a temperature range (of 25–45 °C). Where $\Delta {\text{G}}_{{{\text{ads}}}}^{{\text{o}}}$, $\Delta {\text{H}}_{{{\text{ads}}}}^{{\text{o}}}$, and ($\Delta {\text{S}}_{{{\text{ads}}}}^{{\text{o}}}$) are Free energy, the heat of enthalpy, and adsorption entropy, respectively. Hg^2+^ adsorption by DAC@CAH@SK_2_ material was determined $\Delta {\text{G}}_{{{\text{ads}}}}^{{\text{o}}}$ parameter was calculated from the following equations Eqs. (15), (16), and (17). The plotting of ln KC vs (1/T) temperature in Kelvin for the Hg^2+^ adsorption onto the DAC@CAH@SK_2_ composite is presented in Fig. 15.

15 $${\text{K}}_{{\text{C}}} = \frac{{{\text{C}}_{{{\text{ad}}}} }}{{{\text{C}}_{{\text{e}}} }}$$

16 $${\Delta \text{G}}_{{{\text{adsn}}}}^{{\text{o}}} = - {\text{RT ln K}}_{{\text{C}}}$$

17 $${\text{ln K}}_{{\text{C}}} = \frac{{{\Delta \text{S}}_{{{\text{adsn}}}}^{{\text{o}}} }}{{\text{R}}} - \frac{{{\Delta \text{H}}_{{{\text{adsn}}}}^{{\text{o}}} }}{{\text{RT }}}$$

As Kc, Cad, and Ce are a thermodynamic equilibrium constant, the Hg2+ concentration taken by DAC@CAH@SK2 material at equilibrium (mg/g), and the Hg2+ concentration at equilibrium (mg/L), respectively. While R is the universal gas constant.

As presented in Table 6, the negative values of $\Delta {\text{G}}_{{{\text{adsn}}}}^{{\text{o}}}$ and $\Delta {\text{H}}_{{{\text{ads}}}}^{{\text{o}}}$ demonstrate that Hg^2+^ adsorption by DAC@CAH@SK_2_ is spontaneous and exothermic, respectively. While the arrangement increasing and disorder lowering occurrence was proved from the negative value of ΔS^o^_ads_.

**Table 6:** Thermodynamic parameters for the adsorption of Hg^2+^ onto DAC@CAH@SK_2_

System	T (k)	K_c_	ΔG^o^_ads_ (KJ/mol)	ΔH^o^_ads_ (KJ/mol)	ΔS^o^_ads_ (J/mol K)
DAC@CAHSK_2_-Hg^2+^	298	25.36	− 8.00256	− 83.115	− 252.359
	308	8.584	− 5.5055		
	318	3.0956	− 2.98755		

#### Effect of interfering ions and adsorption selectivity

The selectivity parameter is very essential to evaluate the DAC@CAH@SK_2_ composite's adsorption properties^74,75^. Hence, The DAC@CAH@SK_2_ adsorption selectivity for Hg(II) in the presence of different coexisting metal ions was carried out as shown in Table 7. At the Hg(II) adsorption optimum conditions, the DAC@CAH@SK_2_ composite exhibits excellent selectivity for the Hg(II) ions with the interfering metal ions (Ni(II), Zn(II), Pb(II), Cu(II), and Cd(II)) either in binary systems or multiple- components synthetic mixtures. DAC@CAH@SK_2_ exhibits selective adsorption recovery (Re, %) for Hg(II) of 100% with the interference of Cu(II), Ni(II), and Zn(II) ions. While DAC@CAH@SK_2_ shows recovery higher than 97% in the presence of Cd(II) and Pb(II) ions. This excellent adsorption selectivity of DAC@CAH@SK_2_ for Hg(II) is attributed to the presence of abundant nitrogen and sulfur-containing groups such as -NH_2_, -NH^-^ and -SK on the surface of DAC@CAH@SK_2_, which exhibit strong affinity to Hg(II). When Pb(II) coexists with Hg(II), the selectivity coefficient is quite low, which may be illustrated by the hard-soft acid–base theory (HSAB). Both Hg(II) and Pb(II) are sorted as soft ions which have a strong affinity with thiol and nitrogen active groups that present on the DAC@CAH@SK_2_ composite. DAC@CAH@SK_2_ demonstrates outstanding recovery (Re, %) for Hg(II) of 102% and 104% in the presence of the following synthetic mixtures (Hg(II), Pb(II), Cd(II), and Zn(II)) and (Hg(II), Pb(II), Cd(II), Zn(II), and Cu(II)), respectively. As a whole, the DAC@CAH@SK_2_ composite can potentially adsorb and separate Hg(II) in binary and multiple-metal ions systems. Metal sulfides, dithiocarbamates/thiocarbamates, thiosemicarbazones, thioureas, thiadiazole, and thiazoles are a number of agents that have been well identified for the materials' functionalization for Hg(II) adsorption/uptake. Carbon and sulfur groups are the major components of these agents. Because sulfur is very selective towards mercury, its usage in material modification/functionalization is particularly desirable. Furthermore, Hg(II) forms stable complexes with ligands containing nitrogen and oxygen active groups^76^.

**Table 7:** Effect of interfering ions on the recovery of Hg(II) by DAC@CAH@SK_2_ sorbent.

System	Metal ions	Added (µg mL^−1^)	Found (µg mL^−1^)	Re (%)	q (mg/g)	Selectivity Coefficient (α)
Hg(II)–Ni(II)	Hg(II)	150	10.5	100	139.50	∞
	Ni(II)	150	150		0.00	
Hg(II)–Cd(II)	Hg(II)	150	20.26	97.1	129.74	∞
	Cd(II)	150	150		0.00	
Hg(II)–Cu(II)	Hg(II)	150	10.51	100	139.49	77.49
	Cu(II)	150	148.2		1.8	
Hg(II)–Zn(II)	Hg(II)	150	10.48	100	139.52	17.66
	Zn(II)	150	142.1		7.9	
Hg(II)–Pb(II)	Hg(II)	150	13.569	97.8	136.43	4.7
	Pb(II)	150	121		29	
Hg(II), Pb(II), Cd(II), Zn(II)	Hg(II)	150	3.871	104.6	146.13	5.7
	Pb(II)	150	135		15	
	Cd(II)	150	150		0.00	
	Zn(II)	150	139.4		10.60	
Hg(II), Pb(II), Cd(II), Zn(II), Cu(II)	Hg(II)	150	7.4	102	142.60	6.956
	Pb(II)	150	136.5		13.50	
	Cd(II)	150	149.8		0.20	
	Zn(II)	150	143.2		6.80	
	Cu(II)	150	150		0.00	

#### Influence of ionic strength

**Figure 16:**
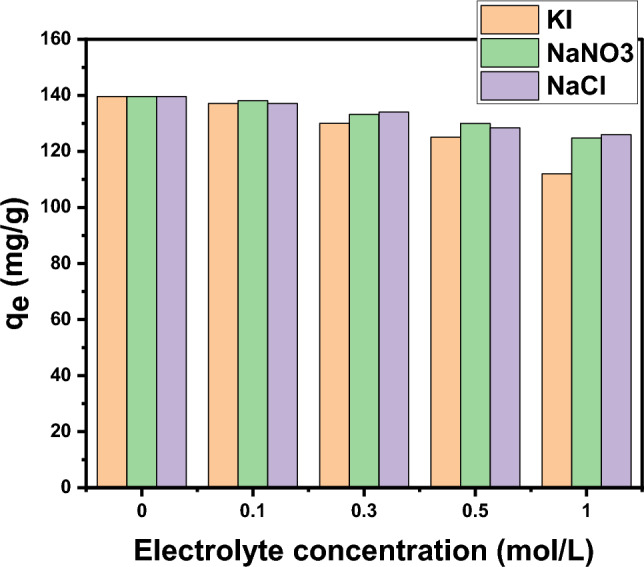
Effect of ionic strength on the Hg(II) adsorption (conditions: 0.01g of DAC@CAH@SK_2_ was taken at 10 ml 150 ppm of Hg^2+^ at pH 6 for 3 h).

The parameter of ionic strength was studied by utilizing Cl^-^, I^-^, and NO_3_^-^ inorganic electrolytes in the form NaCl, KI, and NaNO_3_, respectively. It was investigated by utilizing 0.01 g of DAC@CAH@SK_2_ that was added to 10 ml aqueous solution of 100 ppm of Hg(II), at 25 °C for 180 min and investigated electrolytes concentration range between 0 mol/L and 1 mol/L. As shown in Fig. 16, The DAC@CAH@SK_2_ adsorption efficiency for Hg(II) declined with the investigated electrolytes (Cl^–^, I^–^, and NO_3_^–^) concentration increasing.

#### Desorption and reusability studies

Different eluents were tried for desorption of Hg^2+^ from the DAC@CAH@SK_2_ as ethanol, HCl (0.1 M and 0.2 M), NaOH (0.05 M), NaHCO_3_ (0.1 M), K_2_CO_3_ (0.1 M), thiourea (0.1 M), HNO_3_ (0.1 M), 0.1 M thiourea/0.1 M HNO_3_ mixture (1:1). It was found that thiourea/HNO_3_ mixture (1:1) was the most effective eluent among them and was successfully used for the desorption of the Hg^2+^ from DAC@CAH@SK_2_.

The DAC@CAH@SK_2_ re-using was studied for five sorption–desorption cycles at the optimum conditions with sorption efficiency higher than 89% as shown in Table 8. It was predicted that DAC@CAH@SK_2_ material could be a good sorbent for Hg^2+^ removal from aqueous solutions.

**Table 8:** Repeated adsorption–desorption cycles for DAC@CAH@SK_2_ regeneration by using 0.1 M thiourea/0.1 M HNO_3_ mixture (1:1).

Cycle number	Desorption (%)	Recovery (%)
1	99.12	98.15
2	98.55	97.07
3	94.58	94
4	92.3	91.1
5	90.23	89.18

#### Applications

The application experiments of the DAC@CAH@SK_2_ were obtained by adsorption of Hg^2+^ (100 mg/L and 150 mg/L) from tap water, seawater, and wastewater samples to evaluate prepared chelating agent applicability in real samples. As displayed in Table 9, the Hg^2+^ recoveries (%) from the tested real wastewater samples exceeded 95%. It was demonstrated that DAC@CAH@SK_2_ has remarkable recoveries for the Hg^2+^ that was spiked in the tested water samples proving that the DAC@CAH@SK_2_ can be used for mercury removal from the aqueous environment in actual practice.

**Table 9:** Analysis of spiked Hg^2+^ in real wastewater samples by DAC@CAH@SK_2_ (n = 3).

Wastewater samples type & location	Added (µg mL^−1^)	Found (µg mL^−1^)	qe (mg/g)	Recovery (%)	RSD (%)
Sea water (Marsa Matrouh, Egypt)	0.00	0.00	0.00	0.00	0.00
	50.00	0.45	49.55	99.7	1.17
	100.00	1.02	98.98	99.78	1.21
	150.00	10.3	139.7	100.05	1.25
Tap water (Mansoura university, Mansoura, Egypt)	0.00	0.00	0.00	0.00	0.00
	50.00	0.72	49.28	99.15	1.27
	100.00	1.32	98.68	99.47	1.22
	150.00	10.42	139.58	99.97	1.25
Waste water (Sinbellawin sewage station, Dakahlia,	0.00	0.00	0.00	0.00	0.00
	50.00	0.65	49.35	99.29	1.19
	100.00	1.95	98.05	98.84	1.2
	150.00	17.2	132.8	95.11	1.18

#### Plausible adsorption mechanism

To investigate the possible mechanism of Hg^2+^ adsorption on DAC@CAH@SK_2_, SEM, digital images, and FTIR of the DAC@CAH@SK_2_ and Hg^2+^-DAC@CAH@SK_2_ were evaluated.

##### Digital photographs

**Figure 17:**
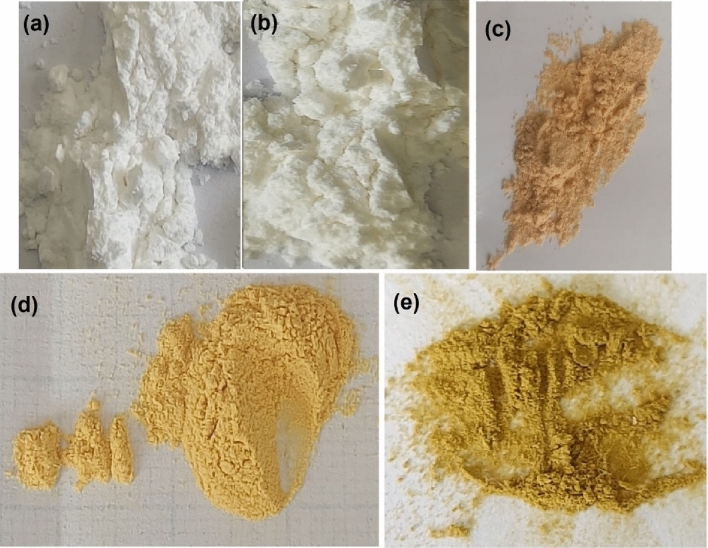
(**a**) native cellulose, (**b**) DAC, **(c)** DAC@CAH, (**d**) DAC@CAH@SK_2_, and (**e**) Hg^2+^-DAC@CAH@SK_2_.

The digital photographs of native cellulose, DAC, DAC@CAH, DAC@CAH@SK_2_, and Hg^2+^-DAC@CAH@SK_2_ were shown in Fig. 17a–e, respectively. An obvious color changes after each step, it converted from the white color of the DAC to the pale sandy fawn color after modification with cyano-aceto hydrazide (Fig. 17b and c) and to dark yellow after modification with Carbon disulfide and potassium hydroxide as in Fig. 17d. The color of the modified cellulose changed into greenish yellow after the adsorption of Hg^2+^ as in Fig. 17e. These changes indicated that the tendency of the modified cellulose towards the adsorption of Hg^2+^.

##### FTIR spectra

**Figure 18:**
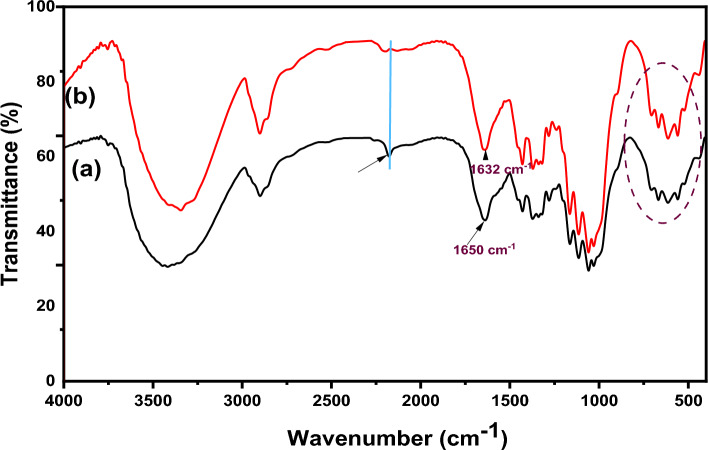
FTIR spectra of (**a**) DAC@CAH@SK_2_ and (**b**) Hg^2+^-DAC@CAH@SK_2_.

In Fig. 18, the DAC@CAH@SK2 composite spectrum before and after Hg^2+^ adsorption is shown^54^. This showed that after Hg^2+^ adsorption the cyano group peak disappeared and C–S between 500 and 600 cm^−1^ became broader than those of DAC@CAH@SK_2_^54^. The azomethane characteristic peak which appears at 1650 cm^−1^ as presented in Fig. 18a showed an obvious shift after the complexation with the Hg^2+^ ions to a lower value of 1632 cm^−1^ as shown in Fig. 18b. Additionally, the bands corresponding to -NH in the DAC@CAH@SK_2_ chelating agent were shifted in the spectrum of Hg^2+^-DAC@CAH@SK_2_. All previous indications led us to estimate that the complexation may be performed by forming a six-ring complex either by the lone pair of the cyano group's nitrogen atom and lone pair of the carbonyl group or through the -NH and a sulfur group of the C–S. The Hg^2+^ complexation with the DAC@CAH@SK_2_ may also carried out through the carbonyl lone pair and the -NH group results in five ring complex formation.

##### Energy-dispersive X-ray spectroscopy (EDS) analysis

**Figure 19:**
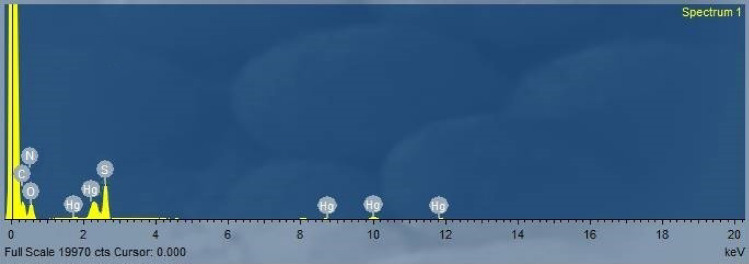
The EDS spectral analysis of DAC@CAH@SK_2_-Hg^2+^.

The Hg(II) adsorption on the prepared chelating agent surface was confirmed and proved through the energy dispersive X-ray (EDS) analysis investigation as presented in (Fig. 19). The appearance of the Hg(II) characteristic peak in the range 1–3 keV demonstrates the Hg(II) adsorption on the DAC@CAH@SK_2_ surface^77^.

Besides the previous evidences, SEM analysis indicates the Hg^2+^ adsorption by the DAC@CAH@SK_2_ composite as the surface of the DAC@CAH@SK_2_-Hg^2+^is brighter than that of DAC@CAH@SK_2_ as shown in Fig. 3c and d which returns to the mercury electric conductivity properties which result in DAC@SAH@SK_2_-Hg^2+^has better electrical conductivity than the DAC@SAH@SK_2_ composite.

**Figure 20:**
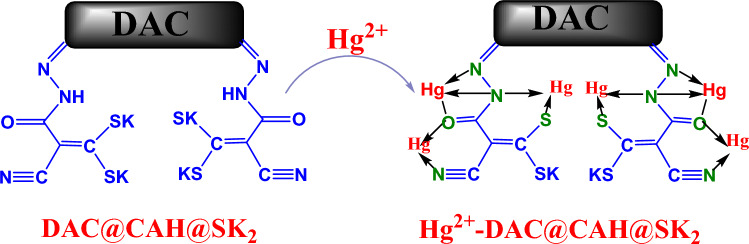
Plausible mechanism of sorption of Hg^2+^ onto DAC@CAH@SK_2_.

The mechanism of any metal ion adsorption onto a material usually is either a chemical reaction or an ion exchange reaction between the chelating agent active groups and the metal ions. Mechanisms applied in Hg(II) adsorption are ion exchange, precipitation, surface-complexation, and complexation/chelation. For thiol (–SH) functionalized adsorbents, the empty orbital of the Hg(II) can bond with free SH lone pair of electrons. Considering the analysis mentioned above, the possible mechanism of Hg(II) ions adsorption onto DAC@CAH@SK_2_ composite could be as shown in Fig. 20. The Hg(II) ions complexes were formed via the coordination bonds with –Sk, C=O, C=N, and lone pair of N–H as presented in Fig. 20 forming five-membered ring and six-membered ring stable complexes^76,78^.

#### Performance of the prepared DAC@CAH@SK_2_

A comparison between DAC@CAH@SK_2_ chelating agent' q_max_ (maximum adsorption capacity) for Hg^2+^ with other adsorbents that were previously used for Hg^2+^ pollutant removal was obtained to improve DAC@CAH@SK_2_ value as shown in Table 10. We can observe that the Hg^2+^ adsorption by the DAC@CAH@SK_2_ is respectably positioned to other researches with a q_max_ of 139.6 at 25 °C since presents a q_max_ value higher than the other reported adsorbents' q_max_. Because of environmental concerns and development requirements, the desorption process and adsorbent regeneration is an essential matter for any adsorbent to evaluate the adsorbent reusing for industrial applications.

**Table 10:** Comparison of adsorption capacity and equilibrium time of various adsorbents for Hg^2+^

Adsorbent	Adsorption capacity (mg g^−1^)	Equilibrium time (min)	Optimal pH	References
MIL-101-Thymine	52	200	6	^79^
AMOF-1′	78	1440	–	^80^
{[Ni1.5(L)(NH_2_-bpy)-(H_2_O)]⋅ 7.5H_2_O}_n_	93.69	300	3	^81^
Chitosan derivatives	122.47	–	2	^82^
Chitosan derivatives	106.4	–	4–6	^83^
Chitosan fluorescent material	108	30	–	^84^
Chitosan fluorescent hydrogel	120.79	40	< 3	^85^
S-SMs	62.3	1500	5.8–8.2	^86^
Zeolites	22.3	–	2	^87^
2CFA-derived zeolite	0.3	1 day	2.5	^88^
Fe_2_O_3_@SiO_2_ thin films	126	45	7	^89^
Bacillus thuringiensis MC28	74	6 days	2–8	^90^
Sulfurized wood biochar	108	30	6	^91^
Polyaniline nanoparticles on the polyurethane foam	15	60	7	^92^
Exhausted coffee waste	32	204	7	^93^
Mercapto-modified bentonite	16	–	6.17	^94^
DAC@CAH@SK_2_	139.6	180	5–8	This work

## Conclusion

**Figure 21:**
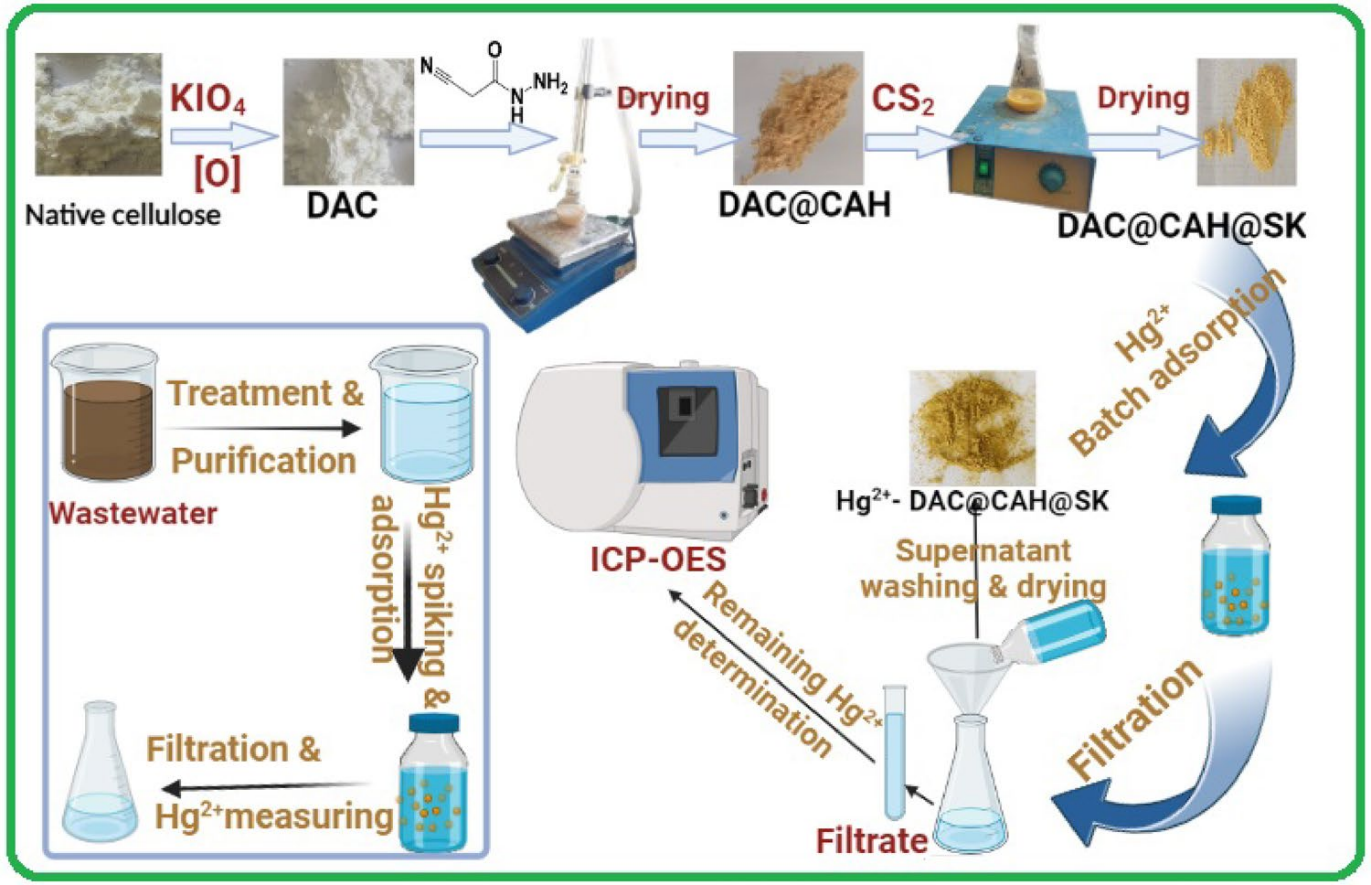
Synthesis of DAC@CAH@SK_2_ and its use for Hg^2+^ adsorption.

We demonstrated the fabrication of the DAC@CAH@SK_2_ composite and its capability to adsorb Hg(II) metal ions from aqueous solutions. The prepared material was fabricated by oxidation cellulose and subsequent chemical modification processes. The prepared materials (DAC, DAC@CAH, DAC@CAH@SK_2_, and DAC@CAH@SK_2_-Hg^2+^) were characterized by different techniques such as SEM, TGA, XRD, FT-IR, CHNS, and BET. The DAC@CAH@SK_2_ exhibited effective and rapid adsorption behavior toward Hg(II) in aqueous solutions, where the optimal initial pH was 7. The adsorption mechanism was revealed by observing the adsorption isotherm and adsorption kinetics. The equilibrated adsorption capacity as a function of the Hg(II) concentration was described precisely using the Langmuir isotherm model, strongly suggesting that the adsorbates form a monolayer with a homogenously distributed surface adsorption energy on the DAC@CAH@SK_2_ surface. The maximal adsorption capacity obtained from the model was 139.6 mg·g^−1^ for Hg(II) ions. Kinetic studies with a pseudo-2^nd^-order kinetic model clearly showed the chemisorption of a single doubly charged mercury ion onto the DAC@CAH@SK_2_ composite. In addition to The negative free enthalpy (ΔG^o^) and enthalpy (ΔH^o^) values indicated that the adsorption of Hg(II) onto DAC@CAH@SK_2_ is spontaneous and exothermic over the investigated temperatures range. The prepared DAC@CAH@SK_2_ composite can be utilized effectively for the selective adsorption and recovery of Hg(II) ions from aqueous solutions. The comparison of q_e_ of DAC@CAH@SK_2_ composite with other sorbents used in Hg(II) removal was reported in the literature. The DAC@CAH@SK_2_ material was applied for the removal of Hg(II) from real waste samples. These results highlight the impact of the chemical surface functionalization of cellulose via oxidation followed by chemical modification toward water remediation. The DAC@CAH@SK_2_ Synthesis and its use for Hg^2+^ adsorption is shown in Fig. 21.

## Data Availability

All data generated or analyzed during this study are included in this published article.
